# Human T-cell leukaemia virus type 1 associated pulmonary disease: clinical and pathological features of an under-recognised complication of HTLV-1 infection

**DOI:** 10.1186/s12977-020-00543-z

**Published:** 2021-01-06

**Authors:** Lloyd Einsiedel, Fabian Chiong, Hubertus Jersmann, Graham P. Taylor

**Affiliations:** 1 Department of Medicine, Alice Springs Hospital, Alice Springs, Northern Territory 0870 Australia; 2 Department of Respiratory Medicine, Faculty of Medicine, Royal Adelaide Hospital, Adelaide, Australia; 3 Department of Infectious Diseases, Faculty of Medicine, Imperial College London, London, UK

**Keywords:** HTLV-1, pulmonary disease, bronchiolitis, bronchiectasis

## Abstract

The lung is one of several organs that can be affected by HTLV-1 mediated inflammation. Pulmonary inflammation associated with HTLV-1 infection involves the interstitium, airways and alveoli, resulting in several clinical entities including interstitial pneumonias, bronchiolitis and alveolitis, depending on which structures are most affected. Augmentation of the inflammatory effects of HTLV-1 infected lymphocytes by recruitment of other inflammatory cells in a positive feedback loop is likely to underlie the pathogenesis of HTLV-1 associated pulmonary disease, as has been proposed for HTLV-1 associated myelopathy. In contrast to the conclusions of early case series, HTLV-1 associated pulmonary disease can be associated with significant parenchymal damage, which may progress to bronchiectasis where this involves the airways. Based on our current understanding of HTLV-1 associated pulmonary disease, diagnostic criteria are proposed.

## Introduction

The human T-cell leukaemia virus type 1 (HTLV-1) is an enveloped, single stranded RNA, human deltaretrovirus [1] that was first identified in 1980 [2], and which causes lifelong infection [3]. Worldwide, at least 5–10 million people are infected, predominantly in areas of high endemicity in southern Japan, the Caribbean basin, parts of South America and inter-tropical Africa [4]. An endemic focus is also present in central Australia where infection with the Australo-Melanesian HTLV-1 subtype C (HTLV-1c) is highly prevalent [5]. HTLV-1 is transmitted by cell contact with infected lymphocytes, exposure to which may occur by ingesting breast milk, through condomless sexual intercourse, blood transfusions, organ transplantation and intravenous drug use [3].

Although HTLV-1 preferentially infects CD4+ T cells in vivo, cell entry is achieved via the ubiquitous glucose transporter-1 [6] and neuropilin-1 [7], which permits infection of CD8+ T-cells, monocytes and other lymphoid and non-lymphoid cells [8]. In common with other retroviruses, the HTLV-1 proviral genome is comprised of two long terminal repeat sequences flanking structural, functional and envelope genes. An additional region, termed pX, is located at the 3′ end of the provirus that encodes regulatory proteins, which appear to be critical for viral pathogenesis [3].

Infection with HTLV-1 results in serious health consequences in a minority of those infected [3]. HTLV-1 is thought to be the most oncogenic virus known [9], and is the causative agent of a rapidly progressive haematological malignancy, adult T cell leukaemia/lymphoma (ATL). Two inflammatory diseases, HTLV-1 associated myelopathy (HAM) [10] and HTLV-1 associated uveitis (HAU) [11], are also acknowledged to be caused by HTLV-1 infection. The diagnosis of each of these conditions requires the presence of HTLV-1 infection [10, 12]. The absence of diagnostic criteria for most HTLV-1 associated diseases complicates the assessment of risk for other clinical entities, and few such studies have been attempted [13]. The life time risk of developing an HTLV-1 associated disease is therefore generally given as that of ATL (5%) and HAM (0.25-3.7%) for which data are available [14]. Infection with HTLV-1 is also associated with infective dermatitis [15] and with increased risks of other infectious diseases including tuberculosis [13, 16, 17], complicated strongyloidiasis [18, 19] and crusted scabies [20]. A high number of HTLV-1 infected cells in peripheral blood (HTLV-1 proviral load, PVL) increases risk of HTLV-1 associated complications in cross sectional studies [3, 21–23], and may prospectively predict risk of ATL [24] and HAM [25].

HTLV-1 has long been considered to have a particular tropism for the lung [26, 27], a condition that has been variously termed HTLV-1 associated broncho-pneumonopathy (HAB) [28], HTLV-1 associated bronchiolo-alveolar disorder (HABA) [29] and HTLV-1 associated pulmonary disease (HAPD) [23]. An association between HTLV-1 infection and bronchiolitis was first suggested in 1986 [26]. Lymphocytosis in broncho-alveolar lavage fluid (BALF) was reported from six Japanese patients with HAM the following year [27] and other reports of a T-cell alveolitis quickly followed from Japan [28, 30] and Martinique [31, 32]. Subsequently, the lung was found to have one of the highest HTLV-1 loads in a semi-quantitative analysis of tissue samples from various organs collected at autopsy from a patient with HAM [33]. Notwithstanding the high frequency with which a pulmonary T cell lymphocytosis was reported, few chest X ray abnormalities were found and lung function tests were normal for eight of ten patients studied in Martinique [31]. These studies have been cited to support the subclinical nature of pulmonary involvement by HTLV-1 [27, 30, 31]; however, respiratory symptoms may be masked by the functional limitations imposed by a myelopathy, and the ability of chest imaging and lung function tests to demonstrate small airway involvement at the time of this research was poor [34].

More recent studies suggest that the involvement of the lungs by HTLV-1 is not only relatively common, but can progress to clinically significant disease. In this review we will examine the literature with regards to the clinical and pathological entities that result from HTLV-1 mediated inflammation of the lung, which together comprise what will here be called HTLV-1 associated pulmonary disease (HAPD), and we will use this information to propose diagnostic criteria to further the study of this condition.

## Epidemiological studies

Notwithstanding the frequency with which pulmonary involvement by HTLV-1 has been reported, few studies have attempted to define this risk. An association between HTLV-1 seropositivity and two specific disease entities, diffuse pan-bronchiolitis (DPB) and idiopathic interstitial pneumonia (IIP), was first suggested in 1986 in a hospital based study in an HTLV-1 non-endemic area of Japan [26]. Antibodies to ‘ATL antigen’ were found in eleven of thirteen patients with DPB, eight of thirteen with IIP, but in no patient with clinical asthma or in healthy controls [26]. Subsequent studies in large respiratory services in HTLV-1 endemic areas of Japan found that DPB (unadjusted odds ratio (uOR), 2.76; 95% CI 1.37, 5.57) [35] and cryptogenic fibrosing alveolitis (CFA) (uOR 1.75; 95% CI 1.02, 3.00) [36] were significantly more common among adults infected with HTLV-1 relative to those who were HTLV-1 uninfected.

Community based cohort studies in Japan found an increased risk of self-reported asthma among men in Miyazaki [37] and of any ‘respiratory disturbance’, which included chest X ray findings consistent with previous pulmonary tuberculosis, in men who attended labour law clinical examinations [17]. No association between HTLV-1 infection and respiratory disease was found in a study of 152 adults with HTLV-1 who attempted blood donation in the USA [38]. These studies were done at a time when HTLV-1 PVL could not be routinely measured, and their interpretation is further limited by the older age of participants in one study [37], the use of self-reported diagnoses [17, 37, 38], which may be understated where this could affect employment [17], and the recruitment of working men [17] and blood donors [38] who are less likely to have chronic lung disease.

A recent meta-analysis concluded that the quality of the epidemiological evidence that supports an association between HTLV-1 seropositivity and pulmonary disease was low [13]. This, in part, reflects the varied pulmonary diseases that have been included in these studies and the heterogeneity of their study designs. However, the authors also excluded studies that did not have an HTLV-1 uninfected control group, gave equal weight to findings derived from self-reported and radiologically defined diagnoses and did not include an analysis stratified by HTLV-1 PVL, which is the major risk factor for HTLV-1 associated inflammatory diseases [3, 21–23].

The poor health outcomes resulting from high rates of chronic lung disease among Aboriginal Australians has led researchers in that country to specifically focus on the association between HTLV-1 and pulmonary disease. In central Australia, where the adult prevalence of HTLV-1 exceeds 40% in some Aboriginal communities [39], Aboriginal adults with non-cystic fibrosis bronchiectasis die at a mean age of 42 [40] to 50 [41] years, which is more than 20 years younger than their non-Indigenous peers with this condition [42, 43]. Although alveolar infiltrates are associated with HTLV-1 in central Australia [44], research has focused on chronic airways disease, the extent of which can be more readily quantified radiologically [41, 45].

An association between HTLV-1 and bronchiectasis was first suggested in central Australia in 1993 [46] and this has consistently been demonstrated in subsequent research, which now includes a case series [40], a retrospective cohort study [44], two case-control studies [41, 45], cross-sectional analysis of a hospital-based cohort [47] and a cross-sectional community survey [48]. The risk of bronchiectasis among HTLV-1 seropositive Aboriginal adults was increased approximately two-fold in hospital based, case control studies (adjusted OR (aOR), 1.84; 95% CI 1.19–2.84) [45] (uOR, 2.23; 95% CI 1.89, 3.86) [41]. HTLV-1c infection is also associated with bronchiolitis [47], the characteristic clinical features of which are similar to those reported in Japan [49]. HTLV-1 seropositive Aboriginal adults were nearly three times (aOR 2.9; 95% CI 2.0, 4.3) more likely to have radiologically diagnosed chronic airways disease (bronchiolitis, bronchitis or bronchiectasis) compared to HTLV-1 seronegative controls in a large hospital based cohort [47].

Risk of HAM increases exponentially when HTLV-1 PVL exceeds 1000 HTLV-1 DNA copies per 10^5^ peripheral blood mononuclear cells (PBMC) [21] and chronic airways disease in Aboriginal adults with HTLV-1 is also strongly associated with a high HTLV-1 PVL in peripheral blood. In a recent hospital based, case control study that controlled for other causes of bronchiectasis including childhood lower respiratory tract infections (LRTI), risk of bronchiectasis increased 1.07 times with every 100 unit increase in HTLV-1 DNA copies per 10^5^ peripheral blood leukocytes (PBL) and was more than 12-fold higher among Aboriginal adults with an HTLV-1 PVL ≥ 1000 copies per 10^5^ PBL relative to uninfected controls [41]. An analysis of reports of chest imaging in a large case series found more bilateral bronchiectasis among Aboriginal adults infected with HTLV-1 relative to those who were uninfected [40]. In subsequent studies, radiologists, who were blinded to HTLV-1 status, applied a scoring system that included parameters of bronchiectasis severity. Among participants for whom HTLV-1 infection was the only identifiable cause of bronchiectasis, more extensive radiological abnormalities were recorded for those with a high HTLV-1 PVL [41], a phenomenon that was previously demonstrated in a study that included patients with chronic bronchitis [45].

An association between HTLV-1 infection and chronic pulmonary disease was again found in a recent cross sectional study that included 579 residents of remote Aboriginal communities and which assessed pulmonary disease blinded to HTLV-1 status using three separate methodologies. The odds of clinically defined chronic suppurative lung disease, moderate to severe expiratory airflow limitation and radiologically diagnosed bronchiectasis or bronchiolitis among adults with a high HTLV-1 PVL in peripheral blood were increased 7.1 (2.67, 18.74; p < 0.001), 9.8 (3.52, 27.35; p < 0.001) and 14.4 (4.99, 41.69; p < 0.001) times, respectively, in adjusted models [48]. Although social determinants undoubtedly increase the risk of bronchiectasis [40], participants in central Australian studies were from the same communities, making socioeconomic status an unlikely confounder [41, 45, 47, 48].

The clinical significance of HTLV-1 PVL for patients with respiratory disease was further demonstrated during prospective follow up of a hospital-based cohort [47]. Aboriginal adults with a high baseline HTLV-1 PVL (≥ 1000 HTLV-1 DNA copies per 10^5^ PBL) were more likely to die as a result of bronchiectasis than those who were HTLV-1 uninfected or had an HTLV-1 PVL < 1000 copies per 10^5^ PBL [47]. A high HTLV-1 PVL also independently predicted death during follow-up of patients recruited to a recent bronchiectasis case control study [41].

In an HTLV-1 cohort at the UK national referral centre, 14 of 413 (3.4%) patients had CT evidence of bronchiectasis, which was higher than background rates for the UK population [50]. Consistent with an HTLV-1 mediated inflammatory process, risk of bronchiectasis was increased among those with other HTLV-1 associated inflammatory diseases [50]. The prevalence of radiologically confirmed bronchiectasis was far higher among Aboriginal adults with HTLV-1 in a recent community survey in central Australia (12.9%) [48]; however, socioeconomic differences and active case finding in the Australian study make comparisons between studies difficult. Relatively few members of the UK cohort were examined by chest CT (34 of 246 asymptomatic patients; 30 of 167 patients with other HTLV-1 associated diseases), and the cohort includes only a fraction of the estimated 30,000 adults who are thought to be HTLV-1 infected in that country [51]. Moreover, individuals with HTLV-1 associated bronchiectasis may not have been appropriately diagnosed and referred to the UK cohort at the time of this study because HTLV-1 testing has only recently been included in British national bronchiectasis guidelines [52]. A Brazilian case control study, which excluded causes of chronic lung disease other than HTLV-1, reported similar findings [53]. Abnormal results on CT chest were recorded for 22 of 30 patients with HAM, but only four of 18 controls with HTLV-1 who did not have HAM, and bronchiectasis was diagnosed in twelve and two patients with and without HAM, respectively [53].

Whether an increased risk of LRTI contributes to the development of chronic pulmonary disease in people infected with HTLV-1 is unclear. HTLV-1 seropositivity was associated with a two-fold increased risk of infection with *Mycobacterium tuberculosis* in a recent meta-analysis [13]. Japanese adults with HTLV-1 were also more likely to present with pulmonary *Mycobacterium avium-intracellulare* complex infection and had more extensive disease than controls without HTLV-1 who were infected with this non-tuberculous mycobacterium [54]. Although the risk of pneumonia was not increased among adults with HTLV-1 who attempted blood donation in the USA [38, 55] or male workers with HTLV-1 in Japan [17], HTLV-1 seropositive adults admitted to hospitals in HTLV-1 endemic areas of Japan [56] and Australia [44] were more likely to be diagnosed with pneumonia relative to HTLV-1 seronegative individuals. The outpatient setting, selection of healthy individuals and use of self-reported diagnoses may contribute to the lower risk of pneumonia in cohorts of blood donors in the USA [38, 55] and Japanese workers [17]. Interpreting these studies is further complicated by the frequency with which abnormalities on chest imaging are associated with HTLV-1 infection [57, 58], and the lack of stratification of risk by HTLV-1 PVL, higher levels of which may have immunosuppressive effects. For example, the major risk factor for blood stream infections with bacterial pathogens in a case control study that included consecutive Aboriginal patients with HTLV-1 was a high HTLV-1 PVL, a finding that was attributed to impaired host immunity [59]. High HTLV-1 PVL were also recorded for cases of bronchiectasis that were admitted with severe pneumonia prior to the diagnosis of bronchiectasis [41]. However, delays in diagnosis of bronchiectasis are common [60] and the local effects of HTLV-1 mediated pulmonary injury, which will be discussed below, are likely to contribute to risk in this patient group, and the local effects of HTLV-1 mediated pulmonary injury, which will be discussed below, are likely to contribute to risk.

## Clinical studies

A pulmonary lymphocytosis was first described in patients with HAM [31], and the infiltration of HTLV-1 infected cells into the lungs of patients with other inflammatory conditions, including HAU [61] and Sjogren’s syndrome [62], has also been described. This phenomenon reflects the systemic nature of HTLV-1 associated inflammatory diseases and the frequency with which multiple organs can be affected in an individual patient [23, 35, 36, 50]. It is also clear that the lungs may be the principal or sole organ affected [28].

### Radiology

Chest X rays were abnormal for one of six cases in the initial Japanese report [27], and were normal for all 21 patients in the first study from Martinique [31]. An interstitial pneumonitis was apparent in one of ten patients examined by chest computed tomography (CT) in the latter study [31]. Subsequently, a large case series reported abnormal chest X rays, which are most often described as diffuse micro-nodular shadows [30], in half of 126 Japanese patients with HAM [63].

The development of high resolution computed tomography (HRCT) has enabled radiological evidence of pulmonary involvement by HTLV-1 to be studied in more detail. Numerous case reports and small case series have documented HRCT findings consistent with bronchiolitis in adults with HTLV-1 [49, 64–69], which may be extensive and appear as miliary centrilobular nodules accompanied by bronchiectasis [70]. However, most information has been derived from two large Japanese case series that reported chest CT findings in patients with HTLV-1 who did not have other recognised HTLV-1 associated conditions. In the largest such study, abnormal radiological findings were found in 98 of 320 (30.1%) adults examined by chest CT [57]. These were most often centrilobular nodules (29.7%), but other radiological abnormalities including thickening of broncho-vascular bundles (17.2%), ground-glass opacities (15.9%), bronchiectasis (15.6%) and interlobular septal thickening (8.8%) were also apparent [57]. Radiological abnormalities were more frequently observed in a smaller study of 106 Japanese adults with HTLV-1 [58]. Abnormal CT findings were found in 65 (61.3%) patients, including ground-glass opacities (31.1%), bronchiectasis (26.4%), centrilobular nodules (23.6%), and interlobular septal thickening (17.9%) [58] with evidence of pulmonary fibrosis in 7.5% of cases [58]. Centrilobular nodules were also the most common radiological abnormalities found by CT chest examination of adults with HTLV-1 in the USA [71]. These were reported in 28 of 72 patients with ATL, and 6 of 25 patients without ATL, and were accompanied by bronchiectasis in 18 (25%) and 12 (48%) patients with and without ATL, respectively [71]. Interstitial lung disease was also thought to precede the diagnosis of ATL in a Japanese study of 35 consecutive patients [72]. The association between HTLV-1 infection and chronic lung disease is therefore recognised to have implications for staging of ATL [71]. Similar radiological findings have been reported in Brazil where bronchiectasis and centrilobular nodules were detected by HRCT in 14 of 30 patients with HAM but in only three of 18 asymptomatic controls with HTLV-1 [53].

The most common radiological abnormalities identified in adults with HTLV-1 are therefore consistent with chronic inflammation involving the small airways and bronchiectasis. Less common radiological patterns are the interstitial pneumonias, which may be non-specific interstitial pneumonia (NSIP) [58, 73], usual interstitial pneumonia or organizing pneumonias [58]. Cryptogenic fibrosing alveolitis was strongly associated with HTLV-1 in a single study of 72 Japanese patients [36].

Studies that compared the extent of radiological abnormalities for specific disease entities in patients with and without HTLV-1 have found more extensive CFA [36], DPB [35] and bronchiectasis [40] among individuals with HTLV-1. More extensive radiological abnormalities were associated with higher HTLV-1 PVL in peripheral blood in Aboriginal Australians with either bronchiectasis alone [41] or a combination of bronchiectasis and chronic bronchitis [45], and with a higher proportion of CD3+ CD25+ cells in BALF in Japanese adults with CFA [36].

### Histopathology

Histopathological studies of lung samples collected by biopsy or at autopsy are consistent with the radiological patterns reported above. Extensive lymphocyte infiltration involving the large bronchi and respiratory bronchioles [74], particularly in peribronchial areas [75], has been reported in tissue samples obtained at autopsy from patients with HAM. In a case series of six such patients, widespread peribronchiolar and perivascular lymphocyte infiltration was observed with involvement of subpleural spaces and alveoli [76]. High lymphocyte infiltration scores were also recorded for bronchial mucosal glands in large bronchi and this was associated with chronic inflammation and smooth muscle hypertrophy in membranous bronchioles [76]. Lymphocyte infiltration progressing along bronchioles has also been observed in tissue obtained by lung biopsy from patients with HAM [64].

Peribronchiolar lymphocyte infiltration has also been reported from lung biopsies of patients with HTLV-1 who did not have HAM [57, 65, 70, 77, 78]. In a large study that correlated radiological findings and histopathological features of lung biopsy samples for 58 patients without HAM, CT chest findings of centrilobular nodules, thickening of broncho-vascular bundles and ground glass opacities corresponded to lymphocyte infiltration along respiratory bronchioles, and into broncho-vascular bundles and the interstitium, respectively [57]. The potential functional consequences of this phenomenon can be inferred from the partial occlusion of bronchioles due to stenosis [78] and lymphocyte infiltration into the bronchiolar lumen [57, 65, 76]. Notwithstanding the radiological similarities between idiopathic DPB and HTLV-1 associated bronchiolitis, the former can be distinguished histologically by the presence of foamy histiocytes [65]. Another Japanese study reported thickening of alveolar septae due to lymphocyte infiltration in tissue obtained by open lung biopsy from thirteen patients with HTLV-1 who did not have HAM [73]; the major pathological diagnoses recorded were interstitial pneumonias (NSIP, 4; acute interstitial pneumonia, 1; lymphocytic interstitial pneumonia, 1; usual interstitial pneumonia, 1) and bronchiolitis [3] with an organizing pneumonia in a single case [62].

### Lung function

Few large studies have reported the results of lung function tests for adults with HTLV-1, and their interpretation is generally limited by the absence of HTLV-1 uninfected control groups and the inability of conventional lung function tests to reliably detect small airway disease [34], which is the clinical entity most often associated with HTLV-1 in histological and radiological studies [57].

Abnormal lung function has been reported for 13–30% of individuals with HAM. The diffusion capacity to carbon monoxide was reduced for seven of 30 Japanese adults with HAM [30], and two of 15 patients in Martinique had abnormal lung function [31]. In a Brazilian case control study, obstructive (n = 4), and restrictive (n = 5) spirometry results were recorded for nine of 30 patients with HAM, whereas lung function was normal for all asymptomatic controls with HTLV-1 [53].

Among individuals without HAM who have symptomatic pulmonary disease, lung function tests reveal obstructive deficits for individuals with HTLV-1 associated bronchiolitis [49, 77] while a restrictive pattern has been recorded for patients with CFA [36] and organising pneumonia [79]. A mixed obstructive and restrictive picture has been reported for a patient with bronchiectasis [65]. There was no difference in lung function for 46 healthy HTLV-1 seropositive adults who attempted blood donation in the USA compared with 127 HTLV-1 seronegative controls [80].

In the only community based study to date, moderate-severe expiratory airflow limitation was recorded for 30 of 316 adult participants who satisfactorily performed spirometry in remote central Australian Aboriginal communities [48]. The adjusted odds of moderate-severe expiratory airflow limitation were increased nearly ten-fold among participants with a high HTLV-1 PVL relative to uninfected controls. Although such an effect could result from peribronchiolar and intraluminal lymphocyte infiltration, the nature of the underlying lung diseases could not be defined in most cases because chest imaging could not be systematically done in this remote setting [48].

## Pathological basis of pulmonary disease

The strong association between bronchiectasis and a high HTLV-1 PVL [41, 45, 47], the dose–response effect of HTLV-1 PVL on the extent of pulmonary damage [41, 45], and the association between the inflammatory disease HAM and bronchiectasis in HTLV-1 cohorts in the UK [50] and Brazil [53] strongly suggest that HAPD results from an HTLV-1 driven inflammatory process. High prevalences of bronchiectasis and bronchiolitis among otherwise asymptomatic patients with HTLV-1 in Japan [57, 58] and reports of similar radiological patterns in the USA [71], Brazil [53] and Australia [47] indicate that HAPD affects genetically diverse hosts regardless of viral subtype.

1. Broncho-alveolar lymphocytosis is largely comprised of activated T-cells.

A lymphocytosis in BALF has been repeatedly found among patients with HAM [22, 27, 31, 63, 81, 82], but has also been reported in HAU [61], Sjogren’s syndrome [32, 62] and adults with pulmonary involvement who are otherwise asymptomatic [83–85]. This phenomenon is particularly common in the setting of HAM; a BALF lymphocytosis has been reported in 76–83% [22, 31], 67–80% [30, 63] and 82% [82] of patients with HAM from Martinique, Japan and Brazil, respectively. No comparable study, stratified by HTLV-1 PVL, has been done for adults without HAM amongst whom there is a wide range of HTLV-1 PVL [86]. Cells in BALF are predominantly CD3+ CD25+ [35, 36, 85, 87], and HLA-DR+ [85] activated lymphocytes.

A large Japanese study compared PBMCs and BALF cells in four patient groups: (i) HTLV-1 with no HAM (n = 38) (interstitial pneumonia, n = 22), (ii) HAM (n = 8), (iii) HTLV-1 seronegative controls with lung disease (n = 44) (sarcoidosis, n = 15; interstitial pneumonia, n = 10) and healthy volunteers (n = 7) [87]. Activated CD4+ and CD8+ T cells, CD25+ T cells, and CD4+ CD29+ T cells were all significantly increased in patients with HTLV-1 associated pulmonary disease who had HAM relative to HTLV-1 seronegative patients with pulmonary disease and healthy volunteers. Moreover, the proportion of CD25+ T cells in BALF exceeded those in PBMC in most HAM patients, and in many adults with HTLV-1 for whom pulmonary disease was the only clinical manifestation of HTLV-1 infection. The proportion of CD25+ T cells in BALF for patients with HTLV-1 and pulmonary disease without HAM was higher than that of HTLV-1 seronegative adults with lung diseases due to causes other than HTLV-1 [88]. Higher proportions of CD3+ CD25+ cells have also been found in the BALF of patients with HTLV-1 who have DBP [35] and CFA [36] relative to patients with these conditions who are HTLV-1 uninfected.

2. HTLV-1 infected cells infiltrate the lung.

As early as 1989, HTLV-1 DNA was detected in BALF cells, thereby confirming that HTLV-1 infected cells infiltrate into the lungs of patients with HAM [89]. Subsequently, higher HTLV-1 PVL were found in PBMCs and BALF from adults with pulmonary inflammation without HAM relative to adults with HTLV-1 who were truly asymptomatic [83, 85, 90]. High proviral loads in BALF cells from patients with and without HAM correlate with, but are generally higher than, those in peripheral blood [81, 85]. Similarly, HTLV-1 tax mRNA, encoding the transcriptional transactivator Tax, was more frequently detected in BALF cells than PBMCs, and this finding was closely associated with the infiltration of activated T-lymphocytes into the lung [91]. The transmission of the virus to HTLV-1 uninfected recipients following lung transplantation from donors with HTLV-1 is therefore not surprising [92].

The difference between HTLV-1 PVL in respiratory secretions and peripheral blood may be even more pronounced when the HTLV-1 PVL in T-cells is compared [93]. HTLV-1 PVL in BALF and PBMCs are generally higher in Japanese patients with the inflammatory disorders HAM [83] and HAU [61] relative to those who are asymptomatic. In a small Japanese study that compared DPB with chronic obstructive pulmonary disease (COPD), HTLV-1 pX DNA was only found in lung tissue from patients with DPB [94]. High local HTLV-1 proviral DNA loads imply the presence of large numbers of HTLV-1 infected cells which are likely to play an important role in the pathogenesis of HTLV-1 associated pulmonary diseases [61]. Moreover, the presence of proviral DNA in BALF is associated with the expression of the HTLV-1 regulatory proteins tax [84, 85, 91, 95], rex [84, 85, 91, 95] and HBZ [84] in BALF, indicating that HTLV-1 infected cells are not passively trafficking through the lung.

3. Pro-inflammatory cytokine and chemokine levels correlate with lymphocyte numbers.

The accumulation of HTLV-1 infected cells in the lungs is associated with an inflammatory milieu in that organ. Levels of cytokines, including IFN-γ and IL-2 [84, 95], and chemokines, such as CCL3 [95, 96], CCL5 [96] and CXCL10 [95], are increased in BALF from patients with HTLV-1 relative to uninfected controls. The autocrine action of IL-2 expressed by HTLV-1 infected T cells increases proliferation and survival of these cells [97]. The chemokines CCL3 and CCL5 are potent chemotactic agents for lymphocytes, which are produced by HTLV-1 infected cells in a Tax inducible manner [98]. CXCL10 is a powerful chemotactic agent for activated CXCR3+ macrophages and lymphocytes that is produced by diverse cell types, including monocytes and epithelial cells, in response to IFN-γ [98, 99].

High concentrations of IFN-γ [95], and the chemokines CCL3 [35, 36, 95, 96] and CXCL10 [35, 36, 95] in BALF, are correlated with higher proportions of lymphocytes [95], including CD3+ HLA-DR+ and CD3+ CD25+ activated lymphocytes [35, 36, 96], and with p40tax expression [95], in BALF. The local production of inflammatory cytokines and chemokines is therefore associated with the infiltration of transcriptionally active HTLV-1 infected lymphocytes into the lungs [95].

Pro-inflammatory chemokines CCL2, CCL5 and CCL20 are also produced by HTLV-1 infected lung epithelial cells in vitro [100], which may contribute to HAPD pathogenesis [98]. Although the regulatory protein Tax has been found in lung epithelial cells from patients with pulmonary disease [100], further studies are required to determine whether HTLV-1 actually infects these cells in vivo. Finally, the accumulation of CD4+ T-cells in BALF from asymptomatic adults with HTLV-1 is associated with high levels of sFasL, a member of the TNF family, in BALF which is thought to block Fas-FasL induced apopotosis, further contributing to the T-lymphocytic alveolitis [101].

Cryptogenic fibrosing alveolitis is the only discrete pathological entity for which the BALF chemokine profile has been studied. In this condition, BALF levels of CCL3 and CXCL10 are higher in patients with CFA who have HTLV-1 relative to those without HTLV-1 infection [36]. Elevated chemokine levels in BALF from patients with CFA are associated with an increased number of activated T cells in BALF [36].

4. Increased cell adhesion molecule expression may facilitate infiltration of HTLV-1 infected lymphocytes.

Increased concentrations of several cell adhesion molecules, including sVCAM, sL-selectin, sP-selectin and sICAM [85], in sera and of sICAM [36, 85] in BALF has been reported in Japanese adults with HTLV-1. Increased ICAM-1 expression by lung epithelial cells may be induced by high levels of IL-1α derived from Tax + cells in the lung [102]. Interactions between these cell adhesion molecules and activated CD25+ T-cells may facilitate the infiltration of HTLV-1 infected cells into the lungs [103, 104].

5. Other possible mechanisms contributing to a pulmonary T cell lymphocytosis.

The clonal proliferation of HTLV-1 infected lymphocytes after exposure to respiratory pathogens might also contribute to a T cell lymphocytosis in BALF with pathogenic potential [40]. While the intra-individual HTLV-1 PVL in peripheral blood is known to remain relatively stable over time [86], there are no longitudinal data for organs to which lymphocytes traffic [103]. Nevertheless, at least in peripheral blood, concomitant infections with other pathogens appear not to be major drivers of HTLV-1 PVL. For example, there is no expansion of HTLV-1 infected clones to suggest selective proliferation of HTLV-1 infected T-cells that are specific to staphylococcal or streptococcal antigens in patients with HTLV-1 associated infective dermatitis, and HTLV-1 PVL does not fall after the treatment of *S. stercoralis* [105]. Consistent with other HTLV-1 associated inflammatory diseases [106], indices of oligoclonality in peripheral blood lymphocytes do not differ between patients with HTLV-1 associated bronchiectasis and those who are asymptomatic [47]. This would not be the case if there was substantial proliferation of HTLV-1 infected clones following recognition of their cognate antigens derived from respiratory pathogens. Further studies are required to exclude the possibility that an HTLV-1 infected lymphocyte sub-population remains resident in the lungs following clonal expansion within that organ.

6. In vitro studies and animal models.

HTLV-1 infects lung epithelial cells in vitro resulting in the expression of HTLV-1 tax and the amplification of proviral DNA [100]. Cytokine, chemokine and cell adhesion molecule mRNA expression was induced by activation of NF-kB and AP-1 when alveolar and tracheal epithelial cells were co-cultured with MT-2 cells in vitro [100]. A tax expressing transgenic mouse model demonstrated increased ICAM and IL-1α expression by epithelial cells and lymphocytes [102], and pulmonary involvement [107] with the presence of Tax protein in lung epithelial cells [100]. Consistent with clinical studies, histopathological examination of lungs from tax transgenic mice revealed a predominantly lymphocytic, inflammatory cell infiltrate in peribronchiolar areas [108]. Inflammatory cytokines, including IL-1β, TNF-α and IFN-γ, and chemokines, including CCL2, CCL3, CCL5 and CXCL10, were detected in the lungs of transgenic mice, but not in control mice [108]. An inflammatory and infiltrative phenotype of HBZ expressing CD4+ T cells was also demonstrated in an HBZ transgenic mouse model [109, 110]. These cells had an enhanced capacity to migrate to the lungs causing pulmonary inflammation that was similar to that of patients with HAPD [109, 110]. Transgenic animal studies therefore suggest that the expression of the regulatory proteins Tax and HBZ may be critical to HAPD pathogenesis.

## A model of HAPD pathogenesis

HTLV-1 associated myelopathy is thought to result from bystander injury as a result of chronic inflammation that is driven by a self-perpetuating, inflammatory feedback loop [111]. Infiltration into the CNS by CCR4+ HTLV-1 infected CD4+ T cells follows their activation by Tax. Secretion of IFN-γ by these Th1-like, HTLV-1 infected, T cells then induces CXCL10 expression by astrocytes, resulting in the recruitment of CXCR3 expressing inflammatory cells, thereby amplifying the inflammatory response [97, 111, 112]. Supporting the central role of CXCL10 in the pathogenesis of HTLV-1 associated inflammatory diseases are observations that high cerebrospinal fluid (CSF) levels of this chemokine predict HAM and the rapidity with which neurological disease progresses [113]. The cytokine and chemokine milieu in individuals with HAPD is remarkably similar to that of patients with HAM. High concentrations of IFN-γ have been recorded in BALF from patients with HAPD [84, 95] and these concentrations correlate with the proportion of lymphocytes in BALF [95]. The concentration of the pivotal, pro-inflammatory chemokine, CXCL10, is high in the affected organs of both conditions [35, 36, 95, 96], and CXCL10 levels are correlated with lymphocyte numbers in CSF and BALF in patients with HAM [112] and HAPD [35, 36, 96, 97], respectively.

**Fig. 1:**
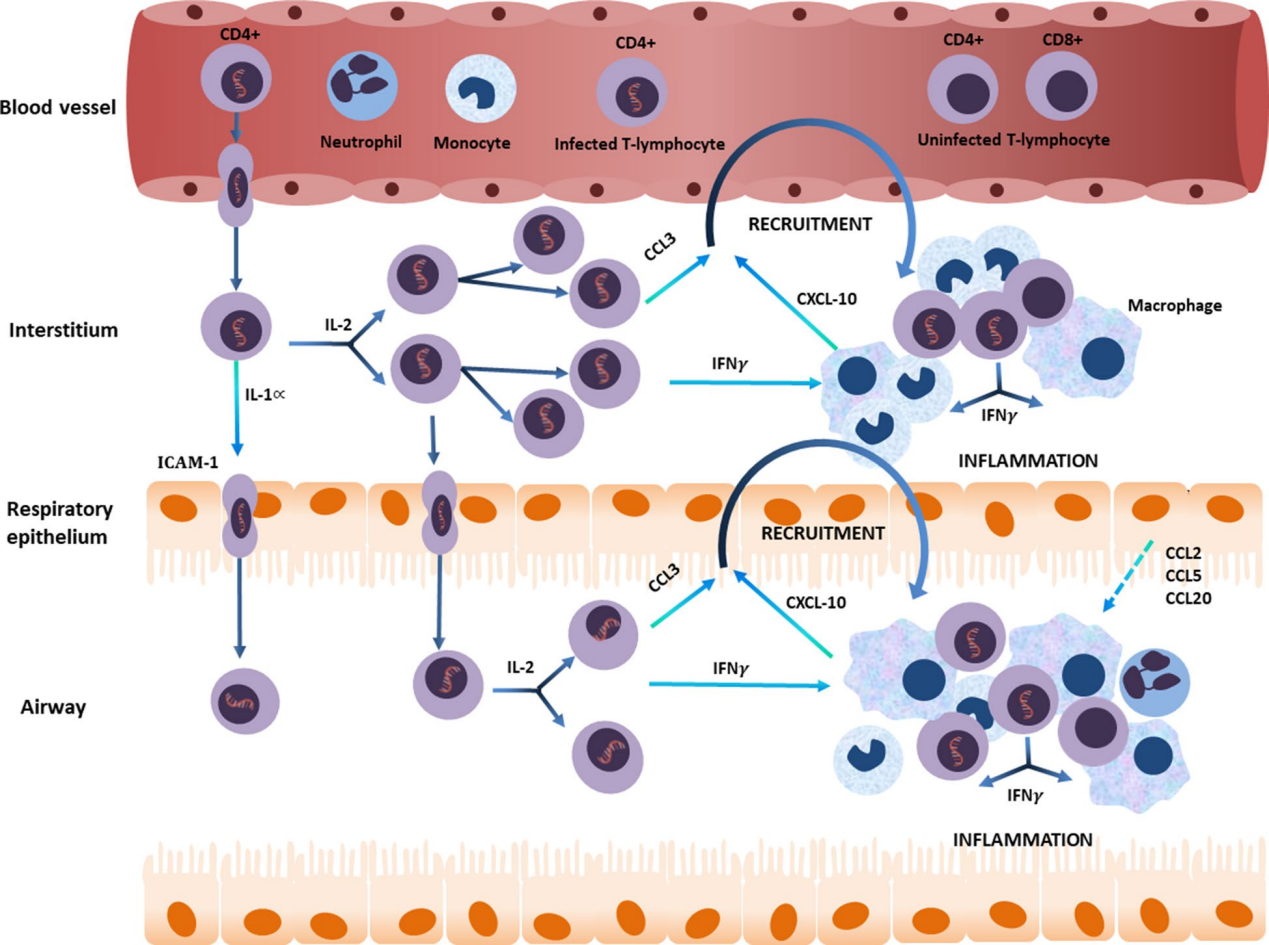
Pathogenesis of HTLV-1 associated pulmonary disease. Proposed model for HAPD pathogenesis. The production of IFN-γ by HTLV-1 infected cells that have infiltrated into the lung induces CXCL10 production by diverse cell types including monocytes and neutrophils, recruiting more CXCR3 expressing, activated T cells and macrophages to the lungs, amplifying the inflammatory process in a positive feedback loop. HTLV-1 infected cells also produce CCL3, which further enhances lymphocyte migration to the area of inflammation, and this is facilitated by increased expression of cell adhesion molecules in response to IL-1α from HTLV-1 infected cells. The production of pro-inflammatory cytokines and chemokines is further augmented by the clonal proliferation of HTLV-1 infected cells in response to IL-2 acting in an autocrine and paracrine manner. Lung epithelial cells may also contribute to this process by producing pro-inflammatory cytokines (dotted line). This inflammatory process involves the interstitium, airways and alveoli, resulting in a lymphocytosis that is detectable in bronchoalveolar lavage fluid, and peribronchiolar lymphocyte infiltration which is apparent histologically

The pathogenesis of HAPD is therefore likely to result from an HTLV-1 driven inflammatory process analogous to that proposed for HAM (Fig. 1). However, relative to the central nervous system, HTLV-1 infected cells may be more likely to infiltrate the lungs, an organ to which large numbers of T cells traffic in support of the immune response to inhaled antigens [103, 114]. We hypothesize that the production of IFN-γ by HTLV-1 infected cells [97] that enter lung tissue induces production of CXCL10 [99], which can be produced by diverse cell types including monocytes and epithelial cells [99]. Recruitment to the lungs of more CXCR3 expressing, activated T cells and macrophages then follows, amplifying the inflammatory effects of these cells in a positive feedback loop (Fig. 1). HTLV-1 infected cells also produce CCL3, increasing migration of lymphocytes to areas of inflammation [98]. This effect may be further enhanced by increased ICAM expression by endothelial and respiratory epithelial cells in response to IL-1α from HTLV-1 infected cells [102]. The production of pro-inflammatory cytokines and chemokines is further augmented by the proliferation of HTLV-1 infected cells in response to high levels of IL-2 derived from these cells [84, 95]. This chronic inflammatory process may finally lead to bronchiectasis, a condition that is the end result of a “vicious cycle” of airway inflammation that is caused by heterogeneous conditions that dysregulate the inflammatory response of susceptible individuals [115, 116].

## Treatment

No directly acting antiviral agents specific to HTLV-1 have been developed and current antiretroviral agents are ineffective in treating chronic HTLV-1 infection [23]. Consequently, therapeutic options for people with HTLV-1 associated inflammatory diseases remain extremely limited. In the absence of strong evidence, several immunomodulatory agents have been used in an attempt to modify the inflammatory response that accompanies the infiltration of HTLV-1 infected cells [23]. The use of prednisolone for HAPD is only supported by case reports of HTLV-1 associated bronchiolitis [49] and organizing pneumonia [117]. A combination of pirfenidone and erythromycin resulted in clinical improvement in a case of interstitial pneumonia [118]. The immuno-modulatory properties of macrolide antibiotics have an established benefit in idiopathic DPB, but have little effect when DPB is associated with HTLV-1 infection [57]. In the only large case control study to date, HTLV-1 associated DPB was less responsive to treatment with macrolides than idiopathic DPB [119]. Similarly, a retrospective study of 46 Japanese patients with HTLV-1 associated DPB who were treated with macrolides found no change in CT appearances at follow up for most patients, with the distribution of centrilobular nodules improving in only seven patients and worsening in four cases [57].

## Proposed clinical criteria for HAPD

Clinical trials are essential to develop therapeutic options for patients with HAPD; however, these are complicated by the absence of criteria that define this condition. To facilitate patient selection for such trials, we therefore propose the following diagnostic criteria:

1. Proven HTLV-1 infection by serology or molecular methods,
2. Radiological evidence of bronchiolitis, bronchiectasis or interstitial pneumonia,
3. The exclusion of other causes including rheumatological diseases and chronic infections with atypical pathogens, such as mycobacteria [52].
4. An HTLV-1 PVL exceeding 1000 copies per 10^5^ mononuclear cells in deep respiratory secretions collected by either sputum induction or bronchoscopy. In resource limited settings, a positive qualitative HTLV-1 PCR test could be used to support a diagnosis of HAPD. However, the predictive value of qualitative HTLV-1 PCR is likely to be significantly lower than that of PVL, and PVL should be arranged for clinical trials.
5. Where histopathological examination of lung biopsy samples is possible, typical features of lymphocyte infiltration into peribronchiolar or interstitial areas should be apparent.

## Conclusions

Clinical and pathological data indicate that the lung is one of several organs affected by HTLV-1 mediated inflammation [23]. Consistent with our understanding of the HTLV-1 associated inflammatory diseases HAM [21] and HAU [12], a high systemic HTLV-1 PVL is a risk factor for HAPD [47]. HTLV-1 associated pulmonary disease is accompanied by infiltration of HTLV-1 infected lymphocytes into the affected organ [83, 85, 90], active transcription of the integrated HTLV-1 provirus in these cells [84, 91, 95, 104] and an inflammatory cytokine/chemokine milieu in BALF [35, 36, 95, 96]. A positive feedback mechanism is likely to underlie the pathogenesis of HAPD, as has been proposed for HAM [111]. Radiological and histopathological studies reveal an inflammatory process involving the interstitium, peribronchial areas and alveoli. Several clinical entities result from this process, including interstitial pneumonias, bronchiolitis and alveolitis, depending on which structures are most affected [57]. In contrast to the conclusions of early case series, HAPD is associated with irreversible parenchymal damage, which may progress to bronchiectasis when the airways are affected.

Pulmonary disease has been reported in association with HTLV-1 subtypes A and C in genetically diverse populations in Japan [28, 29], the Caribbean [31], Brazil [53], the UK [50], the USA [71] and Australia [40, 41, 44, 45, 47, 48]. The frequency with which HAPD affects individuals with HAM [31, 82] and high rates of HAPD in the only population in which this condition has been systematically studied [41, 45, 47, 48], suggests that HAPD may be a relatively common HTLV-1 associated inflammatory disease. Although an improved epidemiological understanding of HAPD is necessary, the range of clinical entities that comprise this condition presents problems for studies that attempt to define risk. We therefore propose a clinical definition of HAPD that will increase the rigour with which such studies can be performed, and facilitate patient selection for clinical trials.

## Data Availability

Not applicable.

## Abbreviations

HTLV-1: Human T-cell leukaemia virus type 1
HTLV-1c: HTLV-1 subtype C
ATL: Adult T cell leukaemia/lymphoma
HAM: HTLV-1 associated myelopathy
HAU: HTLV-1 associated uveitis
PVL: HTLV-1 proviral load
HAB: HTLV-1 associated broncho-pneumonopathy
HABA: HTLV-1 associated bronchiolo-alveolar disorder
HAPD: HTLV-1 associated pulmonary disease
BALF: Bronchoalveolar lavage fluid
HRCT: High resolution computed tomography
NSIP: Non-specific interstitial pneumonia
CFA: Cryptogenic fibrosing alveolitis
DPB: Diffuse pan-bronchiolitis
IIP: Idiopathic interstitial pneumonia
PBMC: Peripheral blood mononuclear cells
PBL: Peripheral blood leukocytes
LRTI: Lower respiratory tract infections
