# Correction to: Human T-cell leukaemia virus type 1 associated pulmonary disease: clinical and pathological features of an under-recognised complication of HTLV-1 infection

**DOI:** 10.1186/s12977-021-00549-1

**Published:** 2021-02-22

**Authors:** Lloyd Einsiedel, Fabian Chiong, Hubertus Jersmann, Graham P. Taylor

**Affiliations:** 1 Department of Medicine, Alice Springs Hospital, Alice Springs, NT 0870 Australia; 2 Department of Respiratory Medicine, Faculty of Medicine, Royal Adelaide Hospital, Adelaide, Australia; 3 Department of Infectious Diseases, Faculty of Medicine, Imperial College London, London, UK

## Correction to: Retrovirology (2021) 18:1 10.1186/s12977-020-00543-z

Following publication of the original article [1], several typesetting errors were noted by the authors.

In the final paragraph of the “Epidemiological studies” section a sentence fragment was erroneously duplicated, and is highlighted in bold text below:

- However, delays in diagnosis of bronchiectasis are common [60] and the local effects of HTLV-1 mediated pulmonary injury, which will be discussed below, are likely to contribute to risk in this patient group**, and the local effects of HTLV-1 mediated pulmonary injury, which will be discussed below, are likely to contribute to risk.**

Furthermore, the following sentence in the “Histopathology” section incorrectly referred to Ref. [3] and linked to Ref. [62] instead of [73]. The corrections are given in bold below:

- Another Japanese study reported thickening of alveolar septae due to lymphocyte infiltration in tissue obtained by open lung biopsy from thirteen patients with HTLV-1 who did not have HAM [73]; the major pathological diagnoses recorded were interstitial pneumonias (NSIP, 4; acute interstitial pneumonia, 1; lymphocytic interstitial pneumonia, 1; usual interstitial pneumonia, 1) and bronchiolitis **(3)** with an organizing pneumonia in a single case **[73]**.

Finally, the caption for Fig. 1 erroneously included the sentence “Proposed model for HAPD pathogenesis” and has been removed.

The original article has been updated.
